# Serum Metabonomic Research of the Anti-Hypertensive Effects of Ogaja on Spontaneously Hypertensive Rats

**DOI:** 10.3390/metabo10100404

**Published:** 2020-10-12

**Authors:** Dahye Yoon, Bo-Ram Choi, Young-Seob Lee, Kyung-Sook Han, Donghwi Kim, Dae Young Lee

**Affiliations:** Department of Herbal Crop Research, National Institute of Horticultural and Herbal Science, RDA, Eumseong 27709, Korea; dahyeyoon@korea.kr (D.Y.); bmcbr@korea.kr (B.-R.C.); youngseoblee@korea.kr (Y.-S.L.); kshan9@korea.kr (K.-S.H.); kimodh@korea.kr (D.K.)

**Keywords:** metabonomics, NMR spectroscopy, Ogaja *(Acanthopanax sessiliflorus*) fruits, hypertension, spontaneously hypertensive rat

## Abstract

Our previous studies have shown that Ogaja *Acanthopanax sessiliflorus* has an important role in decreasing blood pressure, but its biochemical change characteristic has not been clarified completely at the metabolic level. Therefore, in this study, a combination method of nuclear magnetic resonance (NMR) spectroscopy-based metabonomics and multivariate statistical analyses was employed to explore the metabolic changes of serum samples from spontaneously hypertensive rats treated with Ogaja extracts. In the results of multivariate statistical analysis, the spontaneously hypertensive rat (SHR) groups treated with Ogaja were separated from the SHR group. The group of SHR treated with 200 mg/kg Ogaja was clustered with the positive control (captopril) group, and the 400 and 600 mg/kg Ogaja treatment SHR groups were clustered together. Quantified metabolites were statistically analyzed to find the metabolites showing the effects of Ogaja. Succinate and betaine had variable importance in projection (VIP) scores over 2.0. Succinate, which is related to renin release, and betaine, which is related to lowering blood pressure, increased dose-dependently.

## 1. Introduction

Metabonomics is an emerging field of study and a promising tool for wide area such as disease diagnosis and biomarker discovery. Metabolites are the end products of all cellular processes [1]. Metabolism by internal or external factors can be understood from changes of comprehensive metabolic profiles. Nuclear magnetic resonance (NMR) spectroscopy is a powerful tool for metabonomics study, as it is highly automatable, reproducible, reliable, and fast [2]. Indeed, proton NMR collection times for a single spectrum are often as short as a few minutes.

Hypertension is one of the most common chronic diseases worldwide. According to the World Health Organization (WHO), hypertension is a contributor factor to ischemic heart disease, which is the number one cause of death. Complications including stroke, heart attack, and kidney disease can occur in association with persistent high blood pressure [3]. Therefore, patients with hypertension need life therapy and medication according to the degree of hypertension. Patients with hypertension often rely on medication, but antihypertensive drugs have been reported many side effects [4].

Ogaja is the fruit of *Acanthopanax sessiliflorus* in the Araliaceae family and is widely distributed in Northeast Asian countries [5]. Ogaja is listed as an edible material in the Korean Food Code, and the evidence for safe consumption has already been reported [6]. Ogaja, as an edible fruit, was traditionally used as an ingredient in wine or tea in Eastern Asia. In addition, Ogaja is known to have antiplatelet aggregation activity [7], anti-inflammatory activity [8], and antitumor activity [9].

Our previous studies showed that the ethanolic extracts of Ogaja have an effect on hypertension via vasorelaxation, resulting in decreased blood pressure. Positive results from research on its antihypertensive activity indicate that it could complement existing antihypertensive drugs without side effects [10]. Spontaneously hypertensive rats (SHRs) are a genetic hypertensive model in which 100% hypertension occurs naturally through strict brother-to-sister mating [11]. Blood pressure of SHR significantly increases to 190−200 mmHg after they reach an age of 12 weeks. The cause of hypertension is complicated in SHRs, and they are considered a similar model to human essential hypertension [12].

**Figure 1:**
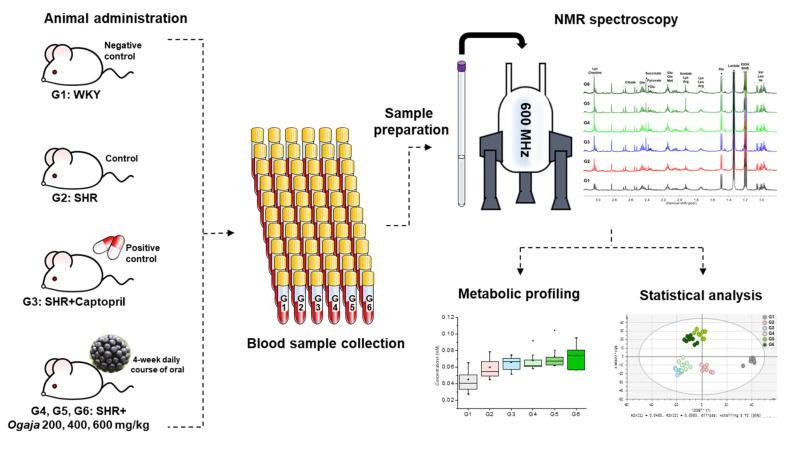
Overall experimental scheme of nuclear magnetic resonance (NMR)-based metabonomics for antihypertensive effects of *Acanthopanax sessiliflorus* fruits (Ogaja) on spontaneously hypertensive rats (SHRs).

In this study, metabolic profiling and metabolic changes in serums of SHRs and Ogaja treated SHRs were analyzed by NMR spectroscopy. Figure 1 shows the overall experimental scheme. Both antihypertensive efficacy and biochemical changes characteristic of Ogaja were explored in our experiment. The results of this paper provide further evidence to understand the potential biomarker for the antihypertensive effect of Ogaja extracts.

## 2. Results

**Figure 2:**
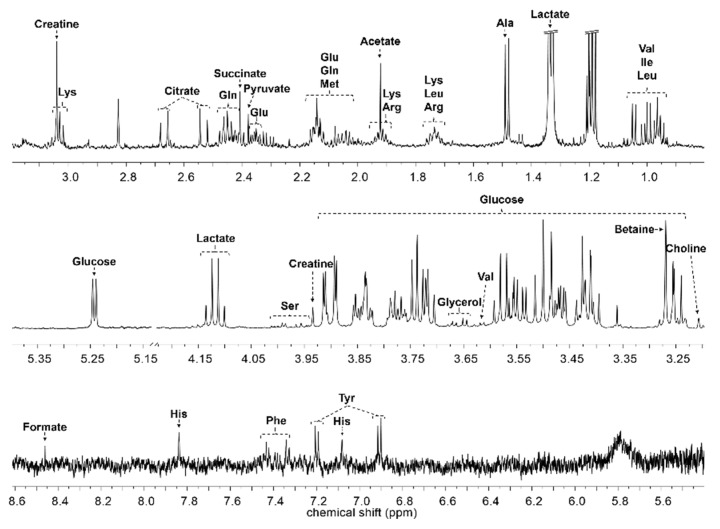
Representative ^1^H nuclear magnetic resonance (NMR) spectrum of rat serum. The major metabolites are annotated on the spectrum.

**Figure 3:**
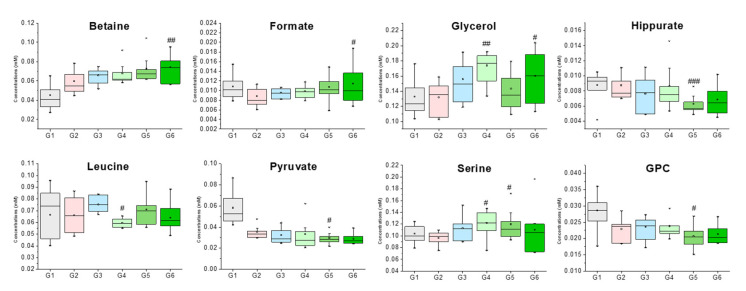
Box plots of metabolites that had groups that were significantly different when compared to G2 (Group 2). ###/##/# indicate significant differences at the *p* < 0.001, *p* < 0.01, *p* < 0.05 levels compared to the G2, respectively.

Serum samples were analyzed using nuclear magnetic resonance (NMR)-based metabonomics to study the antihypertensive effects of *Acanthopanax sessiliflorus* fruits (Ogaja) on rats. Wistar–Kyoto rats (WKYs) were negative control group (G1), G2 were spontaneously hypertensive rats (SHRs) as a control group, and SHRs were treated with captopril (G3), Ogaja 200 mg/kg (G4), Ogaja 400 mg/kg (G5), and Ogaja 600 mg/kg (G6). A representative ^1^H-NMR spectrum of rat serum with annotations of major metabolites is shown in Figure 2. A total of 32 metabolites were identified and quantified in rat serum using Chenomx 600 MHz metabolite database (Chenomx Inc., Edmonton, AB, Canada) and 2D NMR data (Figure S1). Their chemical shifts for identification and concentration data are shown in Table 1. Quantified metabolites were statistically analyzed. Univariate statistical analyses were conducted to determine significantly altered metabolites. Using a *t*-test, the metabolites of G1 exhibited significant differences with other groups (data not shown). However, this result indicates that the WKY is not a suitable control representing the normal blood pressure group in a metabonomics study. It is controversial to use WKY for the control of normal blood pressure group because WKYs are not born under a strict brother-to-sister mating system; therefore, there are genetic differences between WKY and SHR. In this result, the differences of G1 were not significant from a metabolic point of view. Therefore, significant differences with G2 were meaningful in this study. *p*-values that mean statistically significance were calculated for G2, and bar graphs of metabolites whose *p*-values were less than 0.05 are shown in Figure 3.

**Figure 4:**
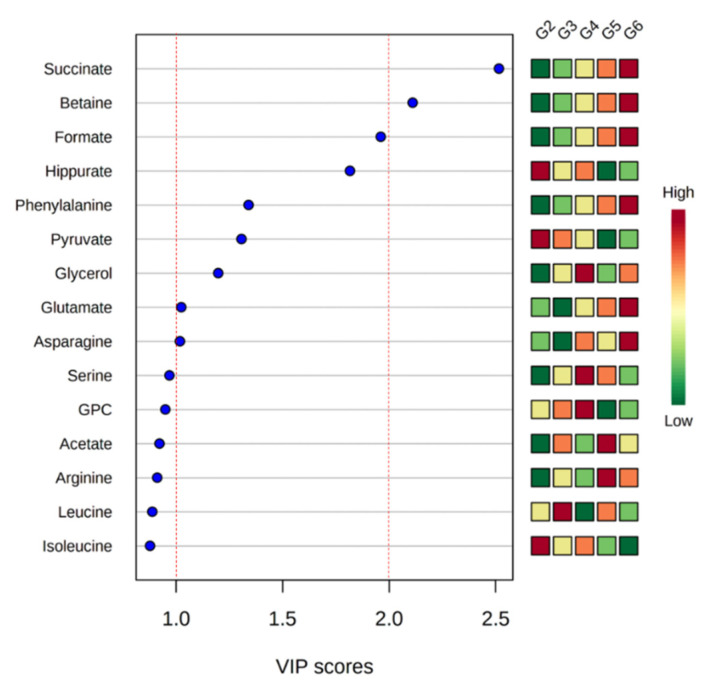
The top 15 metabolites ranked by variable importance in projection (VIP) scores from a comparison of all groups except G1.

Variable importance in projection (VIP) scores of quantified metabolites were calculated to identify meaningful metabolites that showed differences well (Figure 4). All groups except G1 were analyzed to obtain VIP values of metabolites. G1 had a different pattern from other groups, which can affect the VIP scores. A VIP score over 1.0 is typically considered an important metabolite in contributing to the difference [13]. In this result, asparagine, glutamate, glycerol, pyruvate, phenylalanine, hippurate, and formate exhibited VIP scores over 1.0, and betaine and succinate had VIP scores over 2.0.

**Figure 5:**
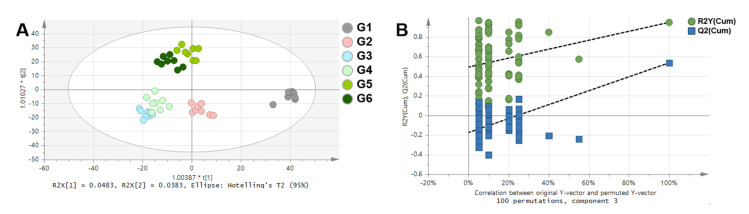
Multivariate statistical analyses of NMR spectra. (**A**) Orthogonal partial least square discriminant analysis (OPLS-DA) score plot of all group comparison (R^2^X = 0.333, R^2^Y = 0.581, Q^2^ = 0.175) (**B**) Validation plot of the OPLS-DA model obtained from 100 permutation tests (R^2^Y-intercept = 0.492, Q^2^Y-intercept = −0.175).

NMR spectra of rat serum samples were analyzed by multivariate statistical analyses to visualize the clustering among groups (Figure 5). Principal component analysis (PCA) was conducted to check the unsupervised distribution of samples (Figure S2). The score plot of PCA showed an unusual cluster independent of the group on the left side. Therefore, these samples were excluded, and eight samples per group were used for the next analyses. Orthogonal partial least square discriminant analysis (OPLS-DA) was additionally performed (Figure 4). OPLS-DA is useful for separating two groups [14]; however, it was used to more effectively check the distribution of the groups in this study. Therefore, in these results, R^2^ and Q^2^ values (goodness of fit and predictive ability of the model, respectively) were low [15]. In the comparison of all groups (Figure 5A), G1 showed a different pattern to the other groups. This result indicates that WKY and SHR have different patterns of metabolic composition. G3 and G4 were clustered and G5 and G6 were clustered. This means that the effects of captopril 100 mg/kg and Ogaja 200 mg/kg on metabolome were similar, and that the effects of Ogaja 400 mg/kg and Ogaja 600 mg/kg on metabolome were similar. Validation plot of the OPLS-DA model was obtained using permutation tests to assess the risk of model over fitting. The permutation test with 100 iterations resulted in Y-intercepts of R^2^ and Q^2^ with values of 0.492 and −0.175, respectively. These data indicated that the model was valid and no over fitting was observed (Figure 5B).

## 3. Discussion

This study was conducted to analyze the antihypertensive effects of *Acanthopanax sessiliflorus* fruits (Ogaja) on spontaneously hypertensive rats (SHRs) using nuclear magnetic resonance (NMR)-based metabonomics.

In our previous study, Ogaja lowered blood pressure dose-dependently, increased renin concentration, and decreased angiotensin-converting enzyme (ACE) activity [10]. Renin converts angiotensinogen to angiotensin I, and ACE converts angiotensin I to angiotensin II in the renin-angiotensin-aldosterone system (RAAS). Renin and ACE act positively in this system, but the concentrations appear negative in SHR [16]. In this study, we tried to determine how Ogaja affects metabolic changes in SHR and how the effect affects hypertension in terms of metabolomics.

Hypertension occurs with many kinds of metabolic changes such as formation of reactive oxidative species in the body [17,18], diabetes, and suppression of the immune system [19]. Captopril, which was used as the positive control in this study, is a single compound, and it effectively lowered blood pressure; however, changes of metabolic profiles were small. Ogaja contained a multi-component and did not lower blood pressure as effectively as captopril, although many metabolic changes occurred. It is thought that the multi-component of Ogaja exerts an antihypertensive effect through the action of improving the negative metabolic effect accompanying hypertension, rather than directly participating in hypotension by acting on a specific target like a drug. Previous study figured out *seco*-triterpenoids in the Ogaja enhanced blood flow and decreased ACE activity [20]. These active compounds in the Ogaja contributed in lowering blood pressure dose-dependently, and they occurred the perturbation of metabolome.

**Figure 6:**
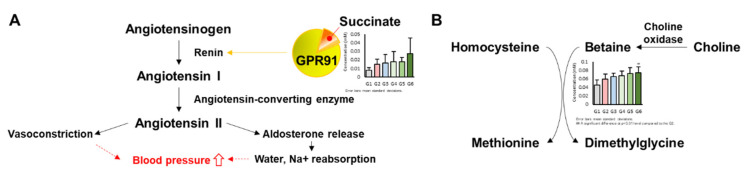
Metabolites that affect regulation of blood pressure. (**A**) Regulation of renin-angiotensin-aldosterone system (RAAS) by succinate and GPR91. (**B**) Methyl donation of betaine to homocysteine.

In these results, Ogaja effected metabolic changes of SHRs. In the variable importance in projection (VIP) analysis, succinate had the highest importance score. It was reported that GPR91, which is a succinate receptor, regulates renin release [21,22]. Increased succinate can regulate the homeostasis of body fluid and blood pressure through the activation of GPR91 [23]. Succinate increased most in G6, and it is thought that blood pressure was reduced by the Ogaja treatment and increased succinate, which maintained homeostasis through renin release (Figure 6A). Renin levels were also higher in the Ogaja treated groups than SHRs in the previous study [10].

Betaine, with the second highest VIP score, was reported to have a negative correlation with blood pressure of dialysis patients [24] and to lower blood pressure [25]. In addition, betaine formed from the oxidation of choline acts as a methyl donor, causing damage to vascular smooth muscle and endothelial cells, converting homocysteine, which can cause hypertension, to methionine [26]. In this result, betaine increased dose-dependently with Ogaja, and in G6 it increased significantly compared to G2 (Figure 6B).

Although no evidence was available that the metabolites were directly involved in or a direct result of lowering blood pressure, lowering blood pressure following Ogaja administration caused meaningful changes in succinate and betaine concentrations. This result suggests that succinate and betaine are potential biomarker candidates for the antihypertensive effect of Ogaja. In addition, metabolic perturbation caused by Ogaja was confirmed, which was a completely different reaction from drug administration as a single compound.

## 4. Materials and Methods

### 4.1. Extraction of Acanthopanax Sessiliflorus Fruits

*Acanthopanax sessiliflorus* fruits (Ogaja) were harvested in Jeongseon, Republic of Korea. A voucher specimen (NIHHS1501) was deposited at the Herbarium of the Department of Herbal Crop Research, National Institute of Horticultural and Herbal Science, Rural Development Administration, Eumseong, South Korea. Ogaja was extracted by reflux extraction with 50% aqueous fermented ethanol at 70 °C for 6 h and extracted again with 50% aqueous fermented ethanol at 70 °C for 3 h. The extract was filtered through a 5 μm filter. The filtered extract was vacuum-concentrated under reduced pressure to obtain 10–20 brix materials. Concentrated extract was sterilized at 80–90 °C for 1 h and lyophilized under reduced pressure (−30 °C, 100 mTorr) for 24 h.

### 4.2. Animal Administration

Adult male Wistar–Kyoto rats (WKYs) and spontaneously hypertensive rats (SHRs) weighing 180 ± 20 g (6 weeks old) were purchased from a commercial breeder (Saeronbio, Inc., Gyeonggi-Do, Korea). We obtained institutional review board approval for this study from the Korea Animal Medical Science Institute (No.15-KE-216). Animals were housed in a controlled vivarium with a temperature of 23 ± 3 °C, humidity of 55 ± 15% and 12 h light/dark cycle with ad libitum feeding of food and water throughout the experiments. WKYs were used as the negative control group (G1), and SHRs were randomly divided into five groups (*n* = 10 per group) as follows: SHR control group (G2), SHR-drug group (G3), SHR-Ogaja 200 mg/kg group (G4), SHR-Ogaja 400 mg/kg group (G5), and SHR-Ogaja 600 mg/kg group (G6). G3 was used as a positive control group, and captopril was used at a dose of 100 mg/kg. Drug and Ogaja administration were conducted using a 4-week daily course of oral administration.

### 4.3. Sample Preparation

Blood samples were collected from the inferior vena cava with a vacutainer tube containing clot activator after anesthetization. Blood samples were kept at room temperature, and serum was obtained using centrifugation at 3000× *g* rpm for 10 min. To carry out nuclear magnetic resonance (NMR) spectroscopy, 500 μL of serum sample was filtered using Amicon Ultra-0.5 (3 kDa cutoff) centrifugal filter (Merck Millipore, Darmstadt, Germany). Then, 200 μL of filtered serum was mixed with 400 μL of 0.2 M sodium phosphate buffer (pH 7.5), which was made with deuterium dioxide. For calibration, the chemical shift on the NMR spectrum and quantification of metabolites, 3-(trimethylsilyl) propionic-2,2,3,3-*d*_4_ acid sodium salt (TSP-*d*_4_) were contained in the buffer. A total of 600 μL sample containing 2 mM of TSP-*d*_4_ was transferred to a 5 mm NMR tube.

### 4.4. NMR Data Acquisition and Data Analysis

Serum samples were measured using a 600.170 MHz Agilent NMR spectrometer (Agilent technologies, Santa Clara, CA, USA). One-dimensional (1D) ^1^H-NMR was performed using PRESAT pulse sequence for the suppression of the water signal. Water presaturation was applied during a 2 s relaxation delay, the acquisition time was 1.703 s, and a total of 128 transients were collected for each sample. Two-dimensional (2D) ^1^H-^1^H correlation spectroscopy (COSY) and ^1^H-^13^C heteronuclear single quantum coherence spectroscopy (HSQC) were also performed to clarify the identification of overlapping metabolites. All NMR data were manually phased and the baselines corrected. Metabolites were assigned and quantified using Chenomx NMR Suite 8.4 Professional (Chenomx Inc., Edmonton, AB, Canada) with the metabolite library database and 2D data. For the quantification of metabolites, the peak of TSP at 0.00 ppm was referred. Each metabolite was manually fitting in Chenomx Profiler, and each concentration was calculated from the concentration of TSP.

### 4.5. Statistical Analysis

A binning process was conducted using Chenomx NMR Suite 8.4 Professional for the multivariate statistical analyses of NMR spectra. The binning area was from 0.5 to 10 ppm with a binning size of 0.001 ppm, and water and ethanol signals were excluded. The binning result was normalized to total area and aligned with the icoshift algorithm of MATLAB (The MathWorks, Natick, MA, USA). The aligned binning result was imported to SIMCA 15.0.2 software (Umetrics, Umeå, Sweden). Principal component analysis (PCA) was conducted to confirm the distribution of unsupervised samples. Therefore, 8 samples for each group were used in the next analyses. Orthogonal partial least square discriminant analysis (OPLS-DA) was performed to visualize group clustering. For the validation of supervised model, 100-time permutation tests were conducted with random shuffling of Y variables.

Quantified metabolites were statistically analyzed using MetaboAnalyst 4.0 (https://www.metaboanalyst.ca). In order to determine the significant difference in metabolic values, Student’s *t*-test was performed. *p*-value for each group was calculated respectively from G2.

## 5. Conclusions

This study was conducted to investigate the hypertensive effect of *Acanthopanax sessiliflorus* fruits (Ogaja) in the serum of spontaneously hypertensive rats (SHRs) using nuclear magnetic resonance (NMR) spectroscopy-based metabonomics. The OPLS-DA score plot showed the separation of Ogaja treated SHR groups from the control SHR group. The Ogaja 200 mg/kg treatment group was clustered with the captopril group (positive control). The Ogaja 400 and 600 mg/kg treatment groups were clustered together. Clustered groups have a similar degree of effects on SHR. The variable importance in projection (VIP) scores were calculated from quantified metabolites. Succinate and betaine showed the highest VIP scores of over 2.0. Succinate is associated with the release of renin and betaine is associated with a lowering of blood pressure. Succinate and betaine may be useful as biomarkers of the antihypertensive effect of Ogaja.

## Figures and Tables

**Table 1:** Identified and quantified metabolites in serum sample from ^1^H-NMR spectra. Values are means (mM) ± standard deviations of concentrations.

Compound	Chemical Shifts (Multiplicities) (ppm)	G1 (mM)	G2 (mM)	G3 (mM)	G4 (mM)	G5 (mM)	G6 (mM)
2-Oxoglutarate	2.43 (t), 3.00 (t)	0.014 ± 0.003	0.018 ± 0.002	0.019 ± 0.005	0.020 ± 0.003	0.018 ± 0.002	0.019 ± 0.004
Acetate	1.91 (s)	0.042 ± 0.019	0.032 ± 0.010	0.038 ± 0.011	0.035 ± 0.009	0.038 ± 0.009	0.035 ± 0.007
Alanine	1.47 (d), 3.77 (q)	0.193 ± 0.027	0.175 ± 0.028	0.169 ± 0.020	0.176 ± 0.024	0.180 ± 0.024	0.154 ± 0.022
Arginine	1.64–1.72 (m), 1.88–1.92 (m), 3.23 (t)	0.078 ± 0.017	0.062 ± 0.025	0.062 ± 0.019	0.063 ± 0.020	0.077 ± 0.025	0.068 ± 0.036
Asparagine	2.85 (dd), 2.93 (dd)	0.023 ± 0.007	0.028 ± 0.011	0.027 ± 0.005	0.031 ± 0.008	0.030 ± 0.006	0.030 ± 0.009
Betaine	3.25 (s), 3.89 (s)	0.045 ± 0.012	0.060 ± 0.012	0.066 ± 0.008	0.068 ± 0.011	0.073 ± 0.014	0.074 ± 0.014 ^##^
Choline	3.19 (s), 3.50 (dd), 4.05 (ddd)	0.010 ± 0.001	0.017 ± 0.002	0.018 ± 0.002	0.019 ± 0.002	0.018 ± 0.002	0.017 ± 0.002
Citrate	2.52 (d), 2.65 (d)	0.102 ± 0.015	0.111 ± 0.012	0.115 ± 0.012	0.116 ± 0.007	0.114 ± 0.016	0.113 ± 0.024
Creatine	3.02 (s), 3.92 (s)	0.050 ± 0.007	0.077 ± 0.011	0.075 ± 0.016	0.068 ± 0.017	0.075 ± 0.012	0.067 ± 0.012
Creatinine	3.03 (s), 4.05 (s)	0.007 ± 0.002	0.006 ± 0.000	0.006 ± 0.002	0.008 ± 0.002	0.006 ± 0.002	0.006 ± 0.001
Formate	8.44 (s)	0.011 ± 0.002	0.009 ± 0.002	0.010 ± 0.001	0.010 ± 0.001	0.011 ± 0.003	0.011 ± 0.004 ^#^
Glucose	3.24 (m), 3.40–3.49 (m), 3.53 (dd), 3.70–3.89 (m), 4.64 (d), 5.23 (d)	3.050 ± 0.223	2.758 ± 0.358	2.583 ± 0.404	2.525 ± 0.398	2.748 ± 0.205	2.717 ± 0.343
Glutamate	2.05−2.12 (m), 2.32−2.35 (m)	0.069 ± 0.010	0.119 ± 0.028	0.118 ± 0.016	0.124 ± 0.010	0.130 ± 0.016	0.123 ± 0.020
Glutamine	2.11−2.14 (m), 2.42−2.46 (m), 3.76 (t)	0.199 ± 0.014	0.230 ± 0.025	0.257 ± 0.028	0.243 ± 0.018	0.241 ± 0.042	0.229 ± 0.021
Glycerol	3.55 (dd), 3.64 (dd), 3.77 (m)	0.133 ± 0.024	0.132 ± 0.022	0.156 ± 0.026	0.174 ± 0.020 ^##^	0.143 ± 0.026	0.160 ± 0.034 ^#^
Glycine	3.55 (s)	0.133 ± 0.013	0.127 ± 0.016	0.136 ± 0.010	0.130 ± 0.010	0.132 ± 0.015	0.123 ± 0.012
GPC	3.22 (s), 3.61 (m), 3.87 (m), 4.32 (m)	0.009 ± 0.002	0.009 ± 0.002	0.008 ± 0.002	0.009 ± 0.003	0.006 ± 0.001 ^#^	0.007 ± 0.002
Hippurate	7.54 (t), 7.63 (t), 7.82 (d)	0.026 ± 0.008	0.030 ± 0.006	0.028 ± 0.004	0.030 ± 0.003	0.032 ± 0.008 ^###^	0.027 ± 0.006
Histidine	3.13 (dd), 3.98 (dd), 7.06 (s), 7.81 (s)	0.005 ± 0.001	0.005 ± 0.002	0.006 ± 0.002	0.005 ± 0.001	0.005 ± 0.001	0.005 ± 0.001
Isobutyrate	1.06 (d), 2.38 (m)	0.041 ± 0.012	0.047 ± 0.007	0.046 ± 0.003	0.047 ± 0.005	0.046 ± 0.006	0.042 ± 0.006
Isoleucine	0.93 (t), 1.00 (d), 1.25 (m), 1.46 (m), 1.97 (m), 3.66 (d)	1.489 ± 0.185	1.694 ± 0.285	1.791 ± 0.385	1.811 ± 0.358	1.582 ± 0.218	1.726 ± 0.363
Lacate	1.32 (d), 4.10 (q)	0.064 ± 0.020	0.067 ± 0.013	0.073 ± 0.009	0.058 ± 0.005	0.071 ± 0.012	0.060 ± 0.017
Leucine	0.95 (t), 1.67–1.74 (m), 3.73 (m)	0.127 ± 0.013	0.119 ± 0.029	0.110 ± 0.012	0.122 ± 0.017 ^#^	0.126 ± 0.020	0.105 ± 0.017
Lysine	1.44–1.51 (m), 1.72 (m), 1.88 (m), 1.92 (m), 3.02 (t), 3.76 (t)	0.021 ± 0.002	0.024 ± 0.004	0.024 ± 0.002	0.024 ± 0.002	0.024 ± 0.002	0.022 ± 0.003
Methionine	2.11 (m), 2.63 (t), 3.85 (dd)	0.020 ± 0.003	0.020 ± 0.004	0.022 ± 0.002	0.022 ± 0.001	0.023 ± 0.003	0.022 ± 0.004
Phenylalanine	7.42 (m), 7.37 (t), 7.32 (dd)	0.058 ± 0.015	0.035 ± 0.006	0.032 ± 0.007	0.033 ± 0.013	0.030 ± 0.005	0.029 ± 0.005
Pyruvate	2.36 (s)	0.104 ± 0.016	0.097 ± 0.011	0.113 ± 0.020	0.122 ± 0.023	0.120 ± 0.026 ^#^	0.110 ± 0.041
Serine	3.84 (dd), 3.94 (dd), 3.98 (dd)	0.008 ± 0.003	0.015 ± 0.006	0.017 ± 0.010	0.018 ± 0.012 ^#^	0.018 ± 0.005 ^#^	0.027 ± 0.018
Succinate	2.39 (s)	0.031 ± 0.005	0.033 ± 0.005	0.031 ± 0.006	0.034 ± 0.007	0.038 ± 0.012	0.031 ± 0.008
Tyrosine	3.05 (dd), 3.19 (dd), 3.93 (dd), 6.89 (m), 7.18 (m)	0.076 ± 0.023	0.083 ± 0.013	0.080 ± 0.008	0.084 ± 0.007	0.084 ± 0.015	0.075 ± 0.010
Valine	0.98 (d), 1.03 (d), 2.26 (m), 3.60 (d)	0.025 ± 0.006	0.031 ± 0.008	0.029 ± 0.014	0.036 ± 0.012	0.027 ± 0.009	0.034 ± 0.013
myo-Inositol	3.27 (t), 3.52 (dd), 3.62 (t), 4.06 (t)	0.029 ± 0.006	0.023 ± 0.003	0.024 ± 0.004	0.024 ± 0.003	0.021 ± 0.004	0.021 ± 0.003

^#^ Significantly different to G2 with *p*-value < 0.05; ^##^ significantly different to G2 with *p*-value < 0.01, ^###^ significantly different to G2 with *p*-value < 0.0.

## Supplementary Materials

The following are available online at https://www.mdpi.com/2218-1989/10/10/404/s1, Figure S1: Representative 2D NMR spectra of SHR serum. 1H-1H correlation spectroscopy (COSY) spectrum (A) and 1H-13C heteronuclear single quantum coherence spectroscopy (HSQC) spectrum (B). Figure S2: Multivariate statistical analyses of NMR spectra. Principal component analysis (PCA) score plot (R^2^X = 0.448, Q^2^ = 0.119).
