# Supplementary material for: Serum Metabonomic Research of the Anti-Hypertensive Effects of Ogaja on Spontaneously Hypertensive Rats
Source: Metabolites. 2020 Oct 12;10(10):404. doi: 10.3390/metabo10100404 (PMC7601199; doi:10.3390/metabo10100404)
Supplement: Supplementary file 1 [file metabolites-10-00404-s001.pdf]

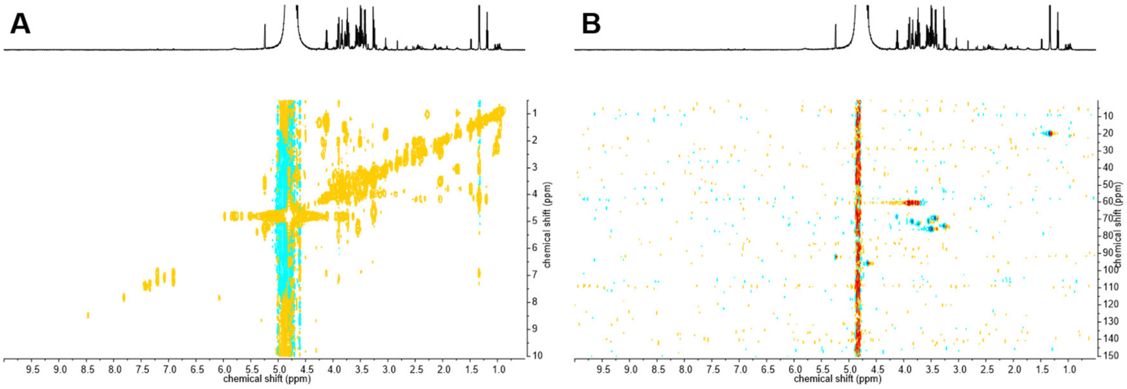

Figure 1. Representative 2D NMR spectra of SHR serum. ^{1}H-^{1}H correlation spectroscopy (COSY) spectrum (**A**) and ^{1}H-^{13}C heteronuclear single quantum coherence spectroscopy (HSQC) spectrum (**B**).

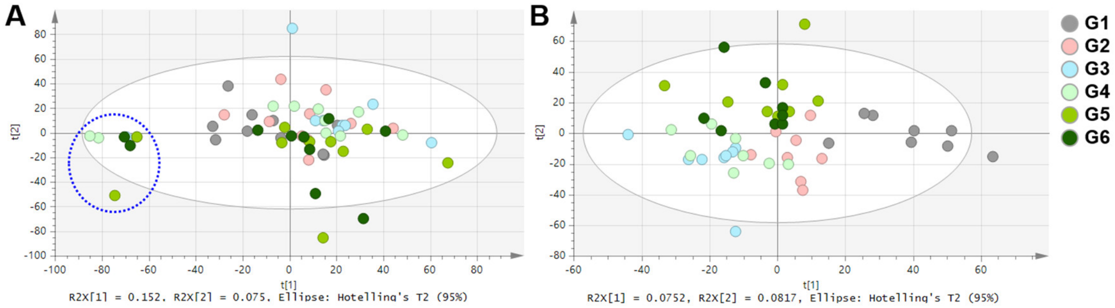

Figure 2. Multivariate statistical analyses of NMR spectra. Principal component analysis (PCA) score plot (R^2X=0.448, Q^2=0.119) (**A**) and partial least squares discriminant analysis (PLS-DA) score plot (R^2X=0.157, R^2Y=0.261, Q^2=0.0838).
